# Orbital reconstruction: titanium mesh implant after excision of orbitozygomaticomaxillary tumors

**DOI:** 10.3389/fvets.2024.1485449

**Published:** 2024-11-21

**Authors:** Elias Wolfs, Graham P. Thatcher, Jason W. Soukup

**Affiliations:** ^1^Dentistry and Oromaxillofacial Surgery, Department of Surgical Sciences, School of Veterinary Medicine, University of Wisconsin-Madison, Madison, WI, United States; ^2^Capital City Specialty & Emergency Animal Hospital, Ottawa, ON, Canada

**Keywords:** computed tomography, 3D printing, virtual surgical planning, orbital reconstruction, titanium mesh

## Abstract

Pathologic lesions of the orbitozygomaticomaxillary complex (OZMC) and caudal oral cavity can be a challenge in veterinary oromaxillofacial surgery. Neoplastic lesions that are in close proximity to or invading the orbit may result in significant loss of structural integrity after curative intent surgery. This in turn may alter the topography of the bulbous oculi (globe) with resultant enophthalmos, diplopia, and entropion. Historically, orbital exenterations have been deemed a suitable option to avoid these complications. However, lesions that do not include the globe in the surgical margin may be overtreated by irreversible orbital exenterations. Orbital reconstruction methods that ameliorate these consequences could be advantageous. A novel approach to reconstruct the orbit with a titanium mesh implant is described and the clinical and ophthalmic outcomes reported.

## 1 Introduction

Orbital exenterations are often needed in cases of larger, infiltrative, malignant neoplasms, especially those involving the orbitozygomaticomaxillary complex (OZMC) (1). The OZMC is the region of the skull where the zygomatic and maxilla bones, as well as bones that contribute to the medial orbital wall (lacrimal, frontal, and palatine bones) unite. The OZMC plays a critical role in the structure, function and esthetics of the skull and orbit contributing to the strength, stability and normal anatomical contours of the facial skeleton. Given the unique intersection of structurally and cosmetically important structures, the proximity of the orbit to the calvarium, and the challenges of access, the surgical excision of OZMC tumors may present unique challenges that make primary surgical goals difficult to achieve. It is generally accepted that the primary goal of any tumor excision is to obtain tumor-free margins. Maintaining proper function, managing local pain, and achieving good to excellent cosmesis are additional goals. The degree to which one may achieve these goals is dependent on location and biological behavior of the tumor as well as the complexity of the resultant reconstruction procedure. Restoring the premorbid structural integrity of the orbit will likely optimize visual function. The alignment and interaction of these structures ensure that light is properly focused, visual information is accurately processed, and vision is maintained.

Neoplastic lesions that invade or are near the OZMC but do not include the bulbous oculi (globe) may require orbital exenterations to achieve clean surgical margins (1, 2). Orbital exenteration may also be performed to avoid ophthalmic complications such as enophthalmos, diplopia, and entropion. This approach could have significant irreversible functional consequences to the patient. Vision of the patient would be limited to one eye, potentially making navigation under photopic and scotopic conditions challenging. Furthermore, if the remaining eye becomes affected by trauma, neoplasia, or any other condition that may result in loss or reduced vision, the patient may become functionally blind.

Exenteration involves the removal of the globe and all soft tissue contents of the orbit. The plane of dissection is outside the extraocular muscles, rather than against the sclera as is performed with an enucleation. This technique is appropriate for neoplastic processes that are contained within the extraocular muscles. If the osseous orbit itself is involved or if the tumor is growing against the bone such that the bone must be removed as a fascial plane to achieve a curative excision, orbitectomy is necessary. The decision to preserve the globe or not and to perform a partial or complete orbitectomy depends on the tumor type, diagnostic imaging (i.e., biological behavior), and client informed consent based on surgical intent and expected outcome. A partial orbitectomy can be either a superior, medial or inferior orbitectomy. A superior orbitectomy involves excision of the frontal bone, exposing the frontal sinus and nasal cavity. An inferior orbitectomy involves the excision of the zygomatic arch +/− portions of the maxilla. Medial orbitectomy is isolated to the region between the superior and inferior orbit and may involve the dorsomedial maxilla, lacrimal, palatine and/or ventral frontal bones. A complete orbitectomy would entail a combination of these (3). With a partial orbitectomy, preservation of the globe may be possible if the globe position is fully supported and stable.

Orbital reconstruction provides support to the globe and averts the irreversible consequences associated with surgical removal. Several orbital reconstruction techniques have been reported in the veterinary literature (4–6). A single case report describes the use of a masseter muscle flap to reconstruct the ventral orbit after a caudal maxillectomy (4). Complications noted after 8 month follow-up were mild facial asymmetry and mild epiphora, which was conservatively managed. A relatively similar approach has been described utilizing a temporalis fascia transposition flap (5). The dog in this report also had an excellent functional and cosmetic outcome but did have mild ventral globe deviation and epiphora 11 months postoperatively. Lastly, in a case series involving four dogs, cerclage wires covered by a prolene mesh and collagen sheet were used to reconstruct the orbit (6). These cases reported good cosmesis and function; however, no objective method of assessment was described. However, no previously published reports have objectively assessed the resultant globe position, which may be an important consideration for prevention of medium-term ophthalmic complications.

In this report, we describe a novel technique for immediate orbital reconstruction with a titanium mesh implant and report surgical outcomes, including an objective immediate and medium-term postoperative measurement of globe position in three dimensions.

## 2 Materials and methods

### 2.1 Case inclusion

Three client-owned dogs were presented to the Dentistry and Oromaxillofacial Surgery Service at the University of Wisconsin-Madison Veterinary Medical Center for assessment and surgical excision of tumors affecting the caudal maxilla and OZMC, which subsequently underwent orbital reconstruction with a titanium mesh implant (Table 1). These cases were included in a previously published report on the maxillary transfacial approach to the midface in dogs (7).

**Table 1:** Case information, diagnosis, surgical procedure, complications and follow-up duration.

Case #	Age (years)	Sex	Breed	Weight (kg)	Tumor location - extent	Pre-operative Diagnosis	Postoperative Diagnosis	Intended Surgical Margin (mm)	Achieved Surgical Margin	Timing of orbital reconstruction	Complications	Follow-up Duration (months)
1	9	FS	Maltese mix	7.2	Right caudal maxillae	Unclassified odontogenic cyst	PSCC	5	Clean	Immediate	Edema	8
2	12	MC	Cocker Spaniel	18.0	Right maxillary 107 - OZMC	APA	APA	10	CleanCleanClean	Immediate	Edema	9
3	11	FS	Bichon mix	15.4	Left maxillary 207–209	CAA	CAA	10	Clean	Immediate	Edema; rostral flap necrosis, oral dehiscence; MRSP infection; ONF revision surgery	6

PSCC, papillary squamous cell carcinoma; APA, amyloid-producing ameloblastoma; CAA, canine acanthomatous ameloblastoma.

### 2.2 Medical records review

Medical records of the three patients were reviewed and the following information was abstracted: history, oral examination results, diagnostic imaging and histopathologic results, surgical planning methods, surgical plan/approach and outcome (Table 1). Follow-up was obtained from medical records.

### 2.3 Diagnostic imaging and virtual surgical planning

Prior to surgery, oncologic staging, including head, cervical, thoracic, and abdominal contrast-enhanced computed tomographic (CT; GE Lightspeed Ultra, GE Healthcare, Milwaukee, WI) imaging, was performed in all cases. Aspirates of mandibular lymph nodes, followed by ultrasound guided aspirates of abnormal (as dictated by imaging results) medial retropharyngeal lymph nodes, were obtained for cytology. Surgical plans for all patients were developed with the aid of virtual surgical planning (VSP) as previously described (8). DICOM files for each patient were imported into a dedicated image segmentation and tri-dimensional (3D) modeling software (Mimics 21.0, Materialize, Leuven, Belgium). A mask of the skull was created using a thresholding operation and a 3D model of the subject skull was created. Osteotomies were planned with margins appropriate for the biological behavior of the lesion in each case, delineated and performed completely in the virtual environment as part of the surgical planning/rehearsal.

### 2.4 3D model printing and custom implant design

**Figure 1:**
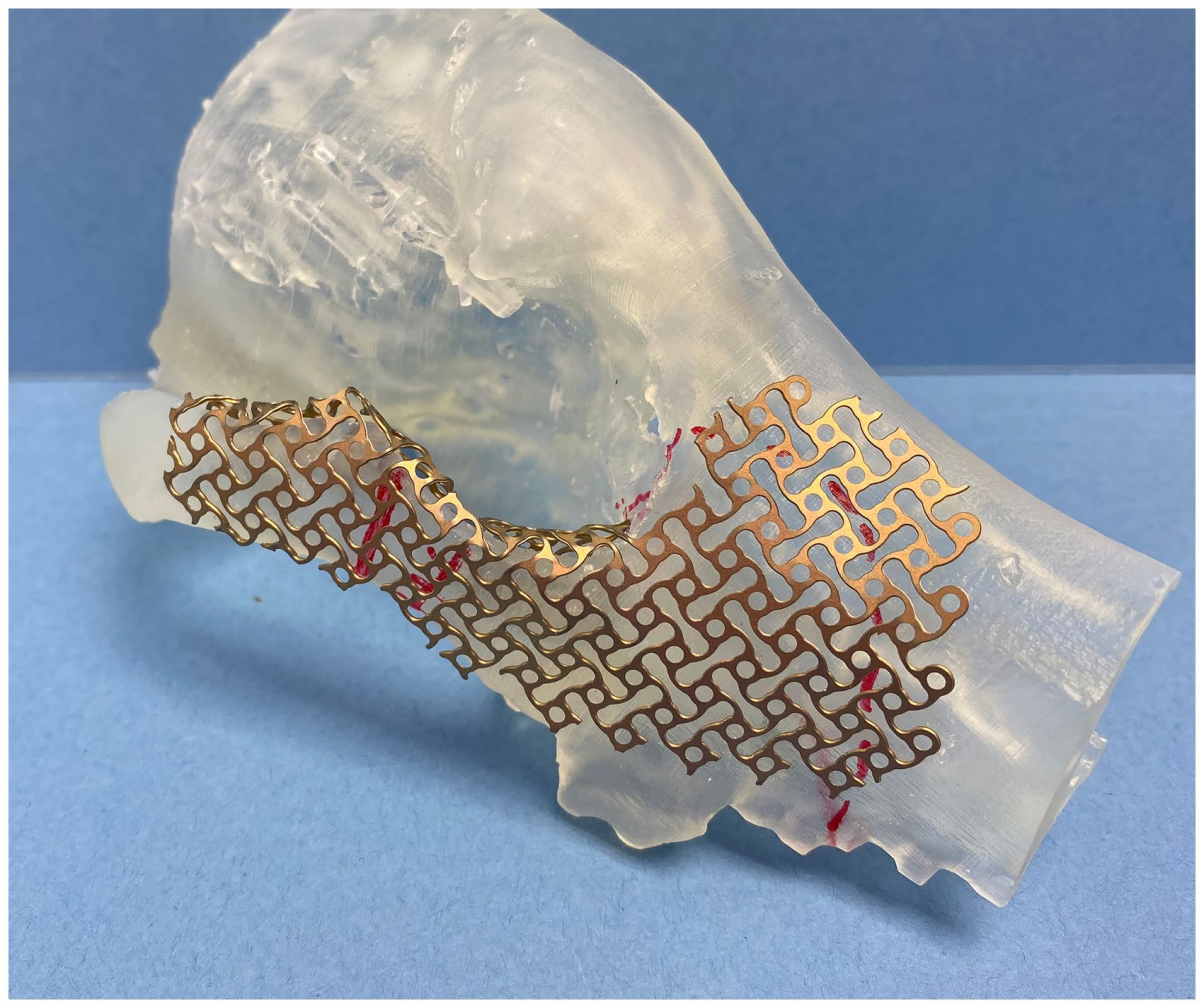
Photograph of a mirrored 3D printed skull with precontoured titanium mesh implant.

In all cases, multiple 3D models were exported into a standard tessellation language mesh and printed to scale (Form 3B, FormLabs, Boston, Massachussets). The mirroring technique was used to represent a simulated normal OZMC of the affected side. Mirrored 3D-printed models were used for the adaptation and contouring of the reconstruction titanium mesh (Low Profile Neuro Plating System; DePuy Synthes CMF, West Chester, PA, USA) (Figure 1). These models were available for review. In one case (case 1), a custom surgical cutting guide was designed (3-Matic, Materialize, Leuven, Belgium) and printed.

### 2.5 Anesthesia and surgical technique

**Figure 2:**
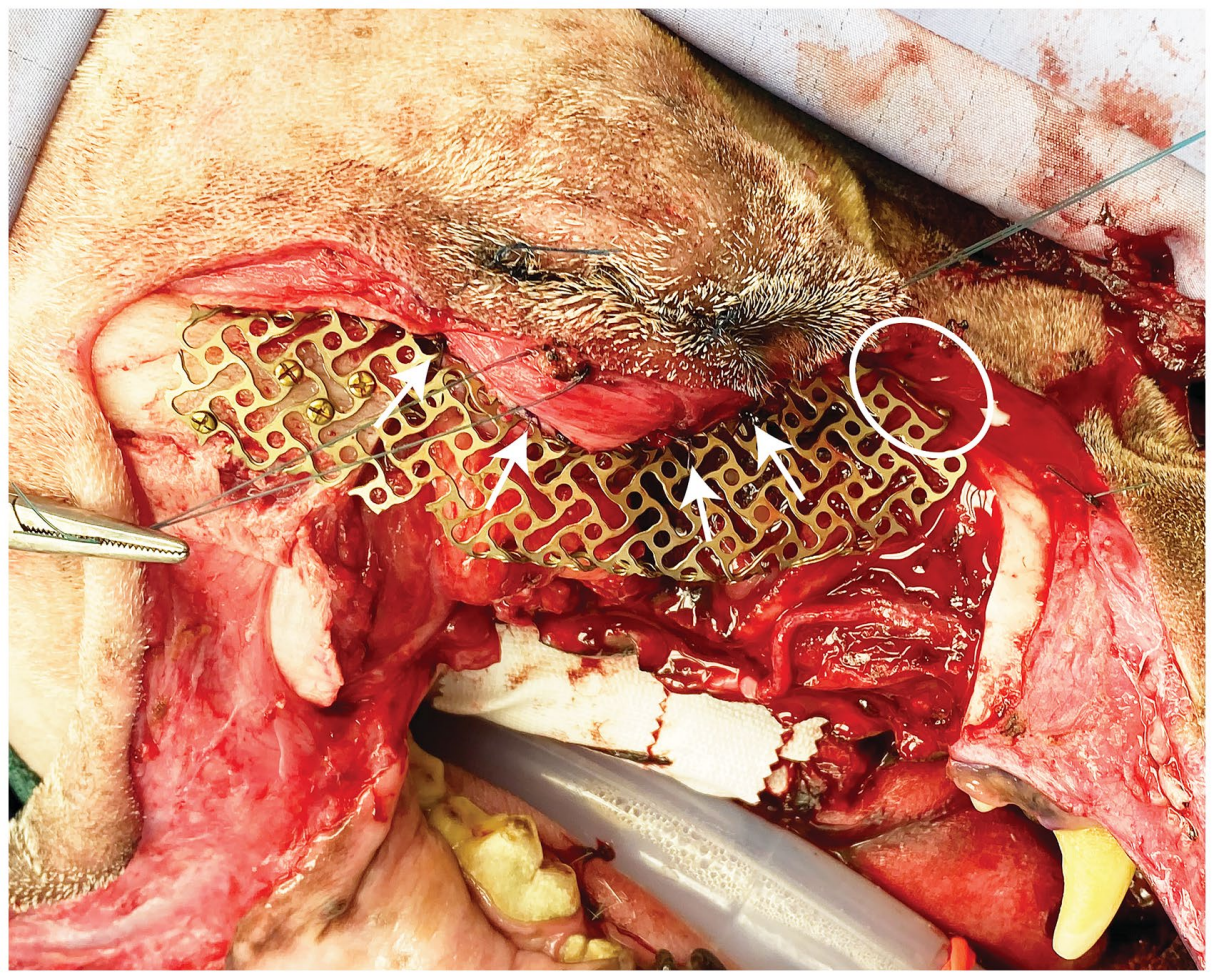
Intraoperative photograph depicting placement of the titanium mesh. Screws can be seen secured to the zygoma and the nasal/maxilla bone (white circle). White arrows highlight sutures securing the periorbita to the implant.

All dogs were placed under general anesthesia within 2–3 weeks after staging for surgical excision using tailored anesthetic protocols as determined by a board-certified anesthesiologist. Standard orotracheal intubation was performed to maintain a secure airway in all patients. Ultrasound guided trigeminal nerve blocks with 0.5% bupivacaine were performed in all patients as previously reported (9, 10). Two patients underwent surgical excision of the lesion by means of a transfacial maxillary approach and one patient by a modified transfacial approach as previously described (7). The periorbita was incised along the medial and ventral orbital rim then reflected dorsal and caudally with a periosteal elevator. In addition, the orbital ligament was incised at its attachment to the zygomatic bone. Osteotomies were made with a piezosurgical unit (Piezosurgery Touch, Mecton, Hilliard OH) as dictated by lesion biological behavior and the surgical plan. In case 1, the printed custom surgical cutting guide was secured with two Kirschner pins to guide the medial orbital and zygomatic osteotomies. Following *en bloc* excision of the neoplastic lesion with predetermined appropriate margins, the pre-contoured, custom-made titanium mesh was trial fitted. If necessary, chairside adaptation by means of minor bending and contouring of the implant was performed prior to securing to the zygomatic and maxilla bones with self-tapping 3–6 mm 1.3 mm titanium cortical screws (Low Profile Neuro Plating System; DePuy Synthes CMF, West Chester, PA, USA) (Figure 2). The periorbita and orbital ligament were secured to the implant with 4–0 monofilament absorbable suture in a simple interrupted pattern (Figure 2). The resultant intraoral defect and titanium mesh implant were closed with a buccal advancement flap. In all cases the extraoral aspect of the transfacial approach was closed routinely.

### 2.6 Postoperative care

All dogs were recovered in the critical care unit (CCU) for administration of IV fluids, tailored pain management protocols via continuous rate infusion (CRI) and continuous pain assessment. Pain management was achieved with tailored multimodal approach in consultation with a board-certified anesthesiologist and/or criticalist. All dogs received application of a cool compress at the surgical site every 4 h until discharge from the hospital. Dogs were administered non-steroidal anti-inflammatory drugs via injection and/or mouth for 3–7 days duration. After 12–24 h in CCU, dogs were transitioned from CRI administration of opioids to either transmucosal or transdermal opioid administration. Dogs were discharged with instructions to return in 10–14 days for assessment and skin suture removal. Different systemic antibiotics were prescribed according to operator preference.

### 2.7 Postoperative globe position

**Figure 3:**
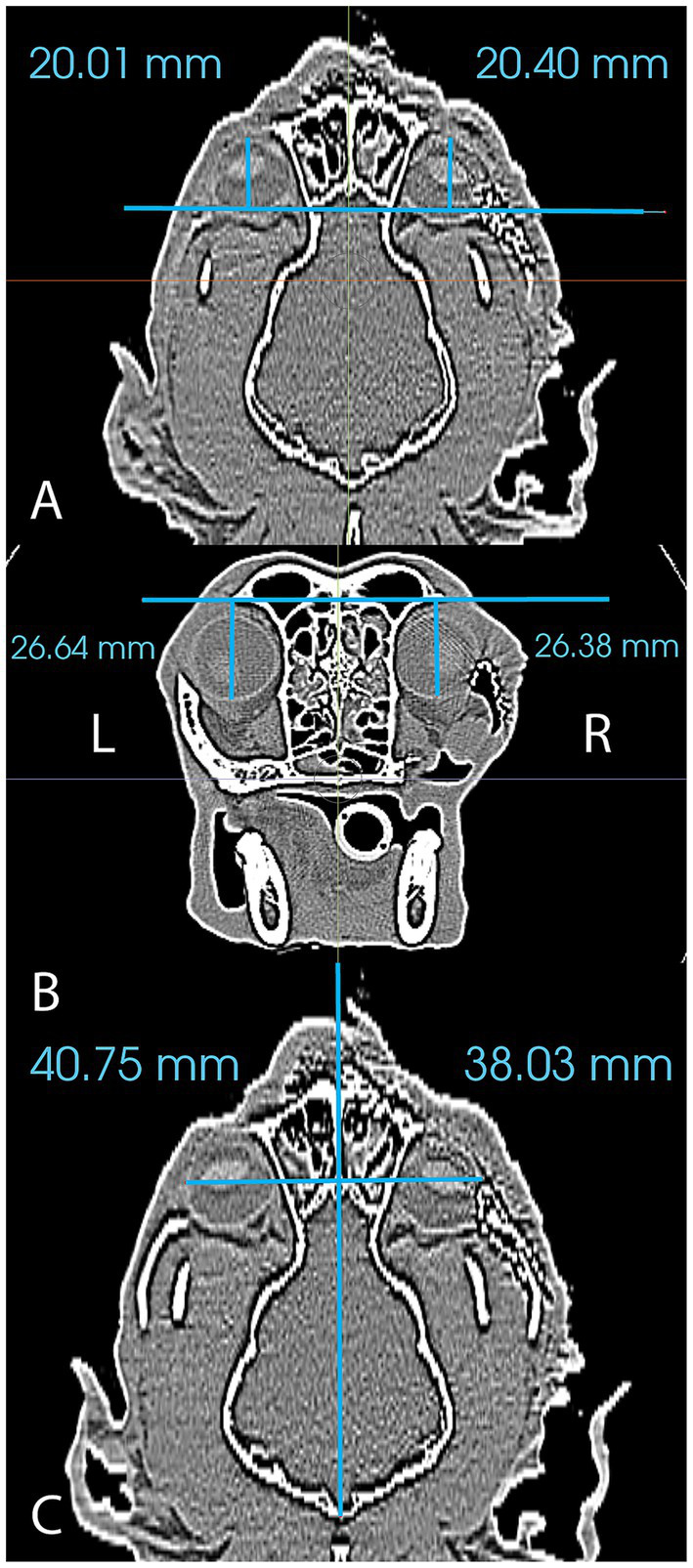
Figure reflecting the method of measurement used to analyze postoperative globe position in the anterior–posterior plane (A), the superior–inferior plane (B) and the lateral-medial plane (C).

Immediate and medium-term (6, 8 or 9 month) postoperative CT (GE Lightspeed Ultra, GE Healthcare, Milwaukee, WI) studies were obtained in all cases to evaluate final mesh and globe position. DICOM files for each patient were imported into a dedicated image viewing software (Invivo 7, Osteoid, Santa Clara, CA) and aligned for symmetry in three axes. Window width and level were adjusted to optimize visualization of the globes and the two-dimensional orientation indicators were centered within the ‘normal’ globe within all (coronal, sagittal and transverse) viewing panels. For the purposes of this study, ‘normal’ was defined as the side opposite the side of orbital reconstruction. Three measurements to define 3D postoperative globe position (anterior–posterior, superior–inferior, lateral-medial) of right and left globes were then obtained (Figure 3). Globe position was determined relative to reference lines drawn between anatomical landmarks (see Table 2 for description). Measurements were obtained for all cases from the immediate postoperative CT and the follow-up CT. The ‘normal’ globe was used as the control for each analysis.

**Table 2:** Descriptions of the measurements used to assess three-dimensional postoperative globe position.

Measurement	Viewing panel	Position within image study	Reference line	Measurement description
Anterior–posterior position	Coronal	First image to contain both globes and zygomas	Line between left and right zygoma; 90° to median plane	From most anterior point of globes to reference line at 90 degrees
Superior–inferior position	Transverse	Center of globe	Line between lateral extent of left and right frontal bones; 90° to median plane	From most ventral point of globes to reference line at 90 degrees
Lateral-medial position	Transverse	Center of globe	Median plane	From most lateral aspect of globes to reference line at 90 degrees

## 3 Results

### 3.1 Case 1

**Figure 4:**
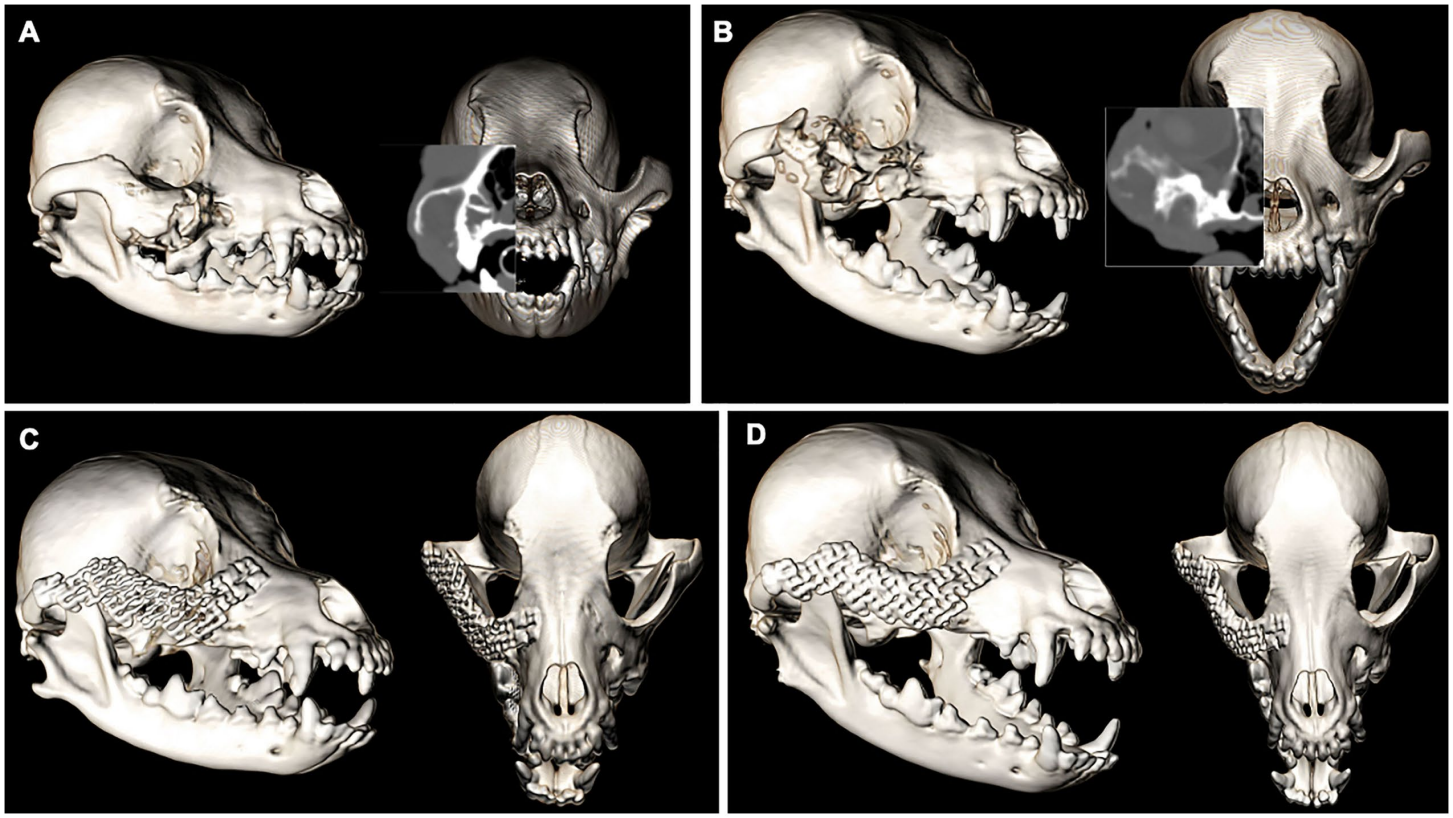
Volume rendered CT images of the skull with reconstructed sagittal plane insert at the time of cyst enucleation (A) and obvious progression of the pathologic process 2 years later (B). The immediate post-op (C) and 8-month follow-up (D). Volume rendered CT images show excellent implant placement and integration and no recurrence of the lesion.

A 9-year-old female spayed Maltese mix was presented for recurrence of a previously enucleated right caudal maxillary odontogenic cyst invading the maxilla, zygomatic and lacrimal bones. The dog initially underwent CT imaging of the head, with cyst enucleation and extractions of the right maxillary third premolar tooth to the second molar tooth 2 years prior (Figure 4A). Due to the suspicious biological behavior of the lesion, full oncologic staging by means of a head, neck, and thoracic CT as well as a repeat biopsy were recommended and pursued at the time of recurrence. The CT revealed progression of the lesion (Figure 4B). The histopathological results were consistent with the initial diagnosis of an odontogenic cyst. Given the multilocular nature of the lesion and concern of failed treatment with conservative cyst enucleation, a more aggressive treatment was recommended. The dog underwent excision of the lesion via a modified transfacial approach with immediate orbital reconstruction. Postoperative CT imaging of the head was performed and revealed excellent excision and implant placement (Figure 4C). The resected tissues were again submitted for histopathological evaluation and revealed to be more consistent with a canine oral papillary squamous cell carcinoma as suggested by immunoreactivity to pan-cytokeratin. The margins were deemed clean albeit narrow (2–3 mm). The dog was presented for suture removal 2 weeks later. Both intra- and extraoral surgical sites revealed no evidence of dehiscence and appropriate continued healing. Repeat imaging was recommended and performed 8 months later. The CT-scan results were consistent with excellent implant integration and no evidence of implant failure as demonstrated by a lack of lucencies around the titanium screws and mesh on CT and the absence of mobility of the implant on clinical palpation. In addition, there was no computed tomographic evidence of recurrence of the previously excised lesion (Figure 4D). Ophthalmic examinations performed at each follow-up visit were within normal limits.

### 3.2 Case 2

**Figure 5:**
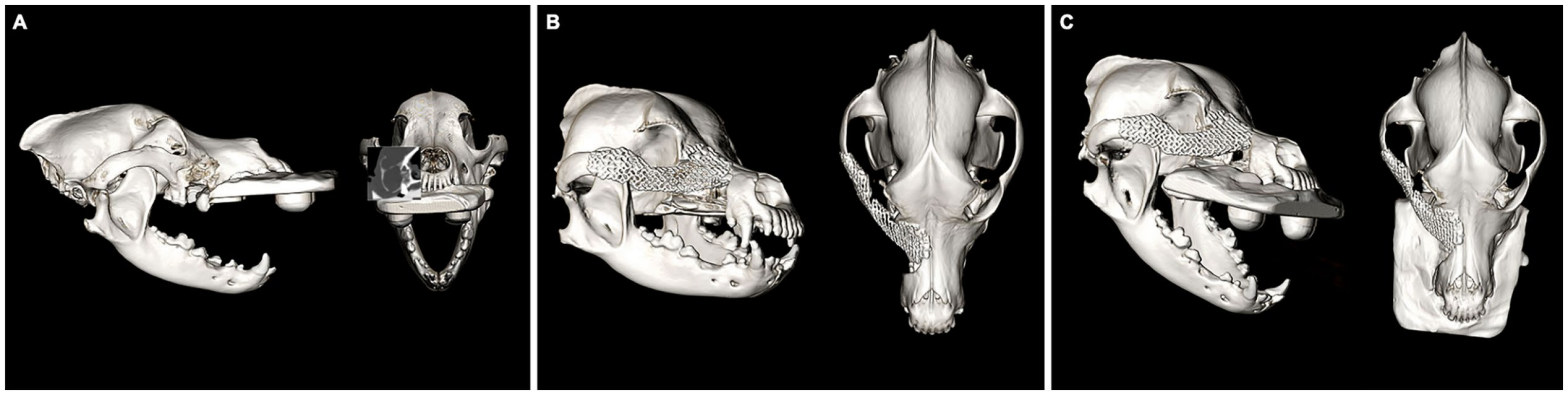
Volume rendered CT images of the skull with reconstructed sagittal plane insert at the time of staging and surgical planning for an APA of the right OZMC (A). The immediate post-op (B) and 9-month follow-up (C). Volume rendered CT images show excellent implant placement and integration.

A 12-year-old male neutered Cocker spaniel was presented for an undiagnosed right maxillary mass. The dog underwent CT imaging of the head, neck, and thorax for staging followed by an incisional biopsy of the lesion for histopathological evaluation and grading. No evidence of metastasis was reported on CT. The expansile, multilobular mass with soft tissue to mineral attenuating and contrast enhancing structures was consistent with the histopathological diagnosis of an amyloid-producing ameloblastoma (Figure 5A). The dog was presented for *en bloc* excision using a transfacial approach with 10 mm margins and immediate orbital reconstruction. The procedure was performed without complications and postoperative CT imaging documented appropriate excision and excellent implant placement (Figure 5B). Histopathological evaluation was in agreement with the previous biopsy result with clean margins. Two weeks later the dog was presented for skin suture removal and healing assessment, which was satisfactory as defined by no evidence of dehiscence and comfortable on extraoral palpation. A nine-month follow-up evaluation was unremarkable, and CT imaging of the head showed excellent implant integration (Figure 5C) and no recurrence of the neoplastic lesion. Ophthalmic examinations performed at each follow-up visit were within normal limits.

### 3.3 Case 3

**Figure 6:**
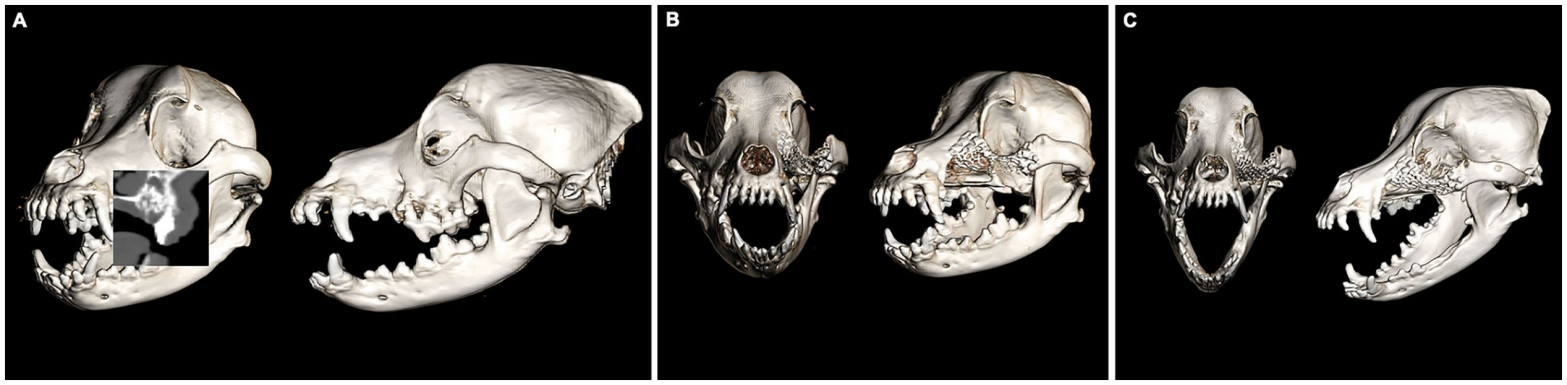
Volume rendered CT images of the skull with reconstructed sagittal plane insert at the time of staging and surgical planning for a CAA of the left OZMC (A). The immediate post-op (B) and 6-month follow-up (C). Volume rendered CT images show excellent implant placement and integration.

An 11-year-old female spayed Bichon mix was presented for a left maxillary canine acanthomatous ameloblastoma (CAA). The dog underwent CT imaging of the head, neck and thorax for surgical planning and staging. The lobular osteolytic, osteoproliferative mineral and soft tissue attenuating, mildly contrast enhancing mass agreed with the histopathological diagnosis of a canine acanthomatous ameloblastoma (Figure 6A). The remainder of the CT examination was unremarkable. The dog underwent surgical excision of the mass with 10 mm margins using the transfacial approach and immediate orbital reconstruction. Postoperative CT imaging of the head showed excellent implant placement (Figure 6B). There were no intraoperative complications, and the histopathological examination was consistent with the previous biopsy report with clean margins. The dorsorostral ~25% of the transfacial flap became necrotic within the first postoperative week and subsequently developed a methicillin-resistant *Staphylococcus pseudointermedius* (MRSP) infection. The necrotic tissue was debrided, and the resultant facial defect was closed with a labial advancement flap. The MRSP infection resolved during a 14–day course of amoxicillin-clavulanate. A 1 × 1 cm oronasal defect developed 6 weeks postoperatively. It was determined that a sharp ventral margin of the underlying titanium mesh was contributing to the defect. In a subsequent surgery, the ventral aspect of the titanium mesh was recontoured to prevent contact with a planned labial advancement flap to close the oral defect, which healed without complication. Repeat imaging was performed 6 months after implant placement. Head imaging with CT revealed excellent implant integration and no evidence of recurrence (Figure 6C). Ophthalmic examinations performed at each follow-up visit were within normal limits.

### 3.4 Post operative globe position

Results of postoperative globe position are reported in Table 3. Globe position measured in the immediate postoperative period was good to excellent in all cases. The postoperative globe position was within 0.5 mm of the control side in the anterior–posterior and the superior–inferior positions. In the lateral-medial position, the globe at the surgical side was within 3 mm of the control. The difference in lateral-medial position in case 1 was 0.08 mm and was 2.72 and 2.79 mm in cases 2 and 3, respectively. More variation in globe position (relative to the control) was noted at the >6 mo post-op evaluation. The surgically treated side in cases 1 and 2 were within 1 mm of the control in all positions. However, in case 3 the difference was between 2.5 and 3 mm in all positions.

**Table 3:** Three-dimensional globe position measurements for cases at the immediate and medium-term postoperative reviews.

Case #	Lateral-medial position			Anterior–posterior position			Superior–inferior position		
	Normal globe	Sx globe	|Difference|	Normal globe	Sx globe	|Difference|	Normal globe	Sx globe	|Difference|
Immediate post-Op measurement (mm)									
1	31.23	31.15	0.08	18.82	18.7	0.12	21.06	21.05	0.01
2	40.75	38.03	2.72	20.01	20.4	0.39	26.64	26.38	0.26
3	38.13	35.34	2.79	13.77	13.89	0.12	23.91	23.95	0.04
Medium-term post-Op measurement (mm)									
1	30.1	29.22	0.88	16.44	16.53	0.09	22.27	22.41	0.14
2	39.39	36.81	2.58	20.25	23.34	3.09	28.44	25.68	2.76
3	29.17	29.14	0.03	17.94	18.4	0.46	21.15	21.33	0.18

## 4 Discussion

The surgical approach and reconstruction described here is a novel technique that may improve the overall outcome for patients with caudal maxillary tumors that involve the OZMC. Historically, these tumor locations were deemed non-resectable or may have been overtreated by means of an orbital exenteration to avoid ophthalmic complications such as diplopia, entropion, corneal ulceration, etc. Alternatively, some may have been treated with globe retention and left to manage potential ophthalmic complications.

Mesh implants are designed for reconstruction of the osseous orbit in humans and to support the placement of grafts. Because of their use in the orbit, these mesh implants are manufactured in the lower-profile 1.5-mm system. The use of titanium mesh for orbital reconstruction in veterinary medicine may be limited to the method described here, as only the medial wall and part of the roof of the orbit in dogs and cats are completely osseous; the lateral wall and floor provide only soft tissue support. It has been reported that the globe in the dog and cat is primarily supported by two fascial structures: the bulbar sheath and the periorbita (11). However, in the authors’ experience, if a significant portion of the ventral orbit is removed, the globe position may shift either ventrally or caudally resulting in complications such as enophthalmos, entropion, and diplopia. Perhaps this is due to the lack of periorbital attachment to an osseous framework (i.e., medial, ventral and lateral orbit). The complication of diplopia may be hard to prove in veterinary patients, but in the human literature it is widely accepted that diplopia compromises quality of life with symptoms such as headache, nausea, dizziness, pain, and blurred vision (12). The current case series utilizing orbital reconstruction suggests sparing of the globe whilst maintaining adequate surgical margins is feasible and offers a similar globe position to the contralateral normal eye.

In the cases presented here the initial histopathological results were all lesion of odontogenic origin. The rationale for immediate reconstruction was based on the biologic behavior of these lesions as well as the predictability of achieving clean margins. It may be judicious to stage the orbital reconstruction for more malignant lesions where the biologic behavior may warrant prior margin assessment. The postoperative histopathological evaluation in case 3 revealed the lesion to be a papillary squamous cell carcinoma even though two previous biopsy results of an unclassified odontogenic cyst were in agreement. Immunohistochemical evaluation of the postoperative lesion had immunoreactivity to pan-cytokeratin (AE1/AE3). Although odontogenic cysts and neoplasms may be immunoreactive for CK19, a component of AE1/AE3, pan-cytokeratin (AE1/AE3) immunoreactivity is more consistent with papillary squamous cell carcinoma than with odontogenic epithelium (13, 14). Fortunately, canine oral papillary squamous cell carcinoma has not yet been reported to metastasize and the margins in the case presented here were clean (15). The authors, however, suggest that a margin check for more aggressive tumor types may benefit from a staged reconstruction procedure. Implant placement in a dirty field would warrant an explant and possible revision surgery with or without radiation treatment or chemotherapy, pending the tumor type.

Our case series supports overall good functional and cosmetic outcome; an improvement over the previously reported temporalis fascia transposition flap or masseter muscle graft where facial asymmetry and globe positioning were altered 8–11 months after surgery (4, 5). In a case series utilizing cerclage wires covered by a prolene mesh and collagen sheet to reconstruct the orbit had a similar outcome as the presented case series here (6).

Overall globe position was very good-excellent in the immediate postoperative period and fair to good at the medium-term follow-up. In the immediate postoperative period, differences in position (compared to the control) were minimal in most dimensions in all cases. The largest difference in orientation was approximately 2.75 mm in the lateral-medial position for cases 2 and 3. In both of these cases, the globe was positioned more medially than the control and would suggest that the contours or the position of the titanium mesh implant slightly decreased the orbital volume. While cases 1 and 3 had excellent agreement between the surgically treated side and the control at the medium-term follow-up appointment, case 2 had displacement values ranging from 2.58–3.09 mm (compared to the control) in all directions. These differences represented a position of the surgical globe that is more superior, medial and posterior than the control and suggests that the contours or the position of the titanium mesh implant decreased the orbital volume. It may be possible to prevent this outcome in future reconstruction procedures with a chair-side adjustment by compressing the reconstructed ventrolateral orbital rim laterally and ventrally by an additional 1–3 mm.

It is difficult to assert the cause of the difference in position between the immediate post-op and the medium-term follow-up measurements in case 2. Potential reasons could include: (1) small differences between the existing orbit and the mirrored (opposite side) orbit used for mesh contouring, (2) postoperative movement of the supporting titanium mesh, (3) inconsistencies in the measurement technique, and (4) early breakdown of periorbital tissue sutures prior to scar tissue formation. We adopted a mirroring technique during the design of the custom-made titanium mesh implant (16). This means that we contoured the implant on a 3D printed model of the mirrored (opposite/normal) orbit. Theoretically, the left and right orbits should approximate mirror images. However, it is possible that the normal orbit in case 2 was not an exact mirror image, which contributed to some loss in orbital volume. Despite our efforts to find a properly validated position measurement technique in the human and veterinary literature, we did not have success. Thus, we resorted to developing our own based on symmetrically isolated anatomical landmarks. It is possible that inconsistencies were present. Prior to any future studies, the measurement system should be validated on a larger population of patients.

A limitation to this study is the lack of a control population of patients that underwent a similar excision without reconstruction of the orbit. However, based on clinical experience, in some cases when orbital reconstruction is not performed, a clinically relevant change in globe position, and the attending complications, is likely. There are no clinical guidelines to predict when this change in position will occur, thus offering reconstruction is prudent, but may not be required.

The authors could not find any known correlation between globe position, relative to a control, and clinical signs of diplopia in the human or veterinary literature. Therefore, any final conclusions must remain largely speculative. However, it seems logical that an increase in the magnitude of globe position discrepancy would correlate with a similar increase in the likelihood of clinical diplopia. While discrepancies less than 2 mm would appear to be a reasonable postoperative outcome, discrepancies approaching 3 mm should raise some concern that the risk of ophthalmic complications may increase. Therefore, case 2 should be followed long-term.

Despite the fact that the titanium mesh implant in case 2 contributed to intraoral dehiscence, it was not attributable to the biocompatibility of the implant but most likely related to the ventral portion traumatizing the overlying advancement flap. Titanium implants have a rough microsurface that supports direct osteointegration when compared with the electropolished stainless steel (EPSS) that is most commonly used in orthopedic implants. Furthermore, titanium owes its superior corrosion resistance to the formation of a thicker metal oxide layer (5–6 nm) compared with EPSS (2–3 nm). Additionally, the equilibrium of a titanium oxide layer is more quickly re-established when it is disrupted compared with a chromium oxide layer. Titanium also has a lower modulus of elasticity (closer to that of bone) than EPSS (17). Moreover, stainless steel implants in the maxillofacial region have been associated with a higher risk of failure (18). Titanium mesh implants have been used historically in the maxillofacial region for severely comminuted fractures (19), cranioplasty to restore the calvarium after excision of neoplastic lesions (20, 21), or as part of the reconstruction of a composite nasomaxillary and superior labial defect (22) in dogs with overall good success.

Reconstruction of the orbit is the standard of care in human maxillofacial surgery (23, 24). Orbital reconstruction involves 6 steps: (1) CT-scan of the patient (with biopsy if no histopathological analysis has been performed), (2) mirroring of the healthy orbit at the affected site in the *in silico* environment, (3) 3D printing of the skull with the mirrored OZMC, (4) adaptation of the customized titanium mesh *ex vivo*, (5) surgical excision of the lesion, and (6) direct or staged insertion of the implant. The latter may be elected if a margin check prior to implantation is deemed necessary.

Virtual surgical planning is the process of planning and rehearsing a surgical procedure completely within the virtual environment on computer models (8). Virtual surgical planning and 3D printing is gaining popularity in veterinary oromaxillofacial surgery as viable tools for the most basic to the most complex cases (8, 25, 26). These techniques can provide the surgeon with improved visualization and, thus, understanding of the patients’ 3D anatomy. Virtual surgical planning is feasible in a clinical setting and may decrease surgical time and increase surgical accuracy (8, 25). Pre-operative implant contouring on a 3D-printed model optimizes anatomic fidelity and can save time during surgery (8). While the process of creating and printing 3D models as well as implant contouring took between 1 and 2 h, it saved intra-operative time and increased anatomical fidelity. Printing of models took between 4 and 6 h but is performed overnight. In addition, the overall process did not delay the time between diagnostic CT acquisition and surgical treatment. The combination of virtual surgical planning and 3D printing were paramount in the successful outcome of the cases presented here.

In this case series, we outline the feasibility of orbital reconstruction after excision of tumors in the caudal maxillae involving the OZMC and orbital support. Careful case selection with appropriate advanced imaging for surgical planning, full oncologic staging to rule out metastasis or potential unrelated comorbidities, and incisional biopsy for histopathology is warranted prior to surgical excision and reconstruction of the orbit. Overall, the cases presented here had a clinically good outcome with adequate structure, function, and excellent cosmesis of the OZMC following oncologic surgery with immediate reconstruction. Although, caution needs to be taken to avoid orbital volume loss.

## Data Availability

The original contributions presented in the study are included in the article/supplementary material, further inquiries can be directed to the corresponding author.

## References

[1] Thomson AE Rigby BE Geddes AT Soukup JW. Excision of extensive Orbitozygomaticomaxillary complex tumors combining an intra- and Extraoral approach with Transpalpebral orbital Exenteration. Front Vet Sci. (2020) 7:569747. doi: 10.3389/fvets.2020.569747 33363228 PMC7759551

[2] Simon GJB Schwarcz RM Douglas R Fiaschetti D McCann JD Goldberg RA. Orbital exenteration: One size does not fit all. Am J Ophthalmol. (2005) 139:11–7. doi: 10.1016/J.AJO.2004.07.041, PMID: 15652823

[3] O’Brien MG Withrow SJ Straw RC Powers BE Kirpensteijn JK. Total and partial Orbitectomy for the treatment of periorbital tumors in 24 dogs and 6 cats: a retrospective study. Vet Surg. (1996) 25:471–9. doi: 10.1111/J.1532-950X.1996.TB01445.X 8923726

[4] Sivagurunathan A Boy SC Steenkamp G. A novel technique for ventral orbital stabilization: the masseter muscle flap. Vet Ophthalmol. (2014) 17:67–72. doi: 10.1111/VOP.12058, PMID: 23710820

[5] Dent B Wavreille VA Selmic LE. Use of a temporalis fascia transposition flap for ventral orbital stabilization after ventral orbitectomy in a dog. Vet Surg. (2019) 48:1058–63. doi: 10.1111/VSU.13162 30677160

[6] Wallin-Håkansson N Berggren K. Orbital reconstruction in the dog, cat, and horse. Vet Ophthalmol. (2017) 20:316–28. doi: 10.1111/VOP.12420 27520591

[7] Thatcher GP Congiusta MC Soukup JW. Application of a maxillary transfacial approach to the caudal oral cavity and orbitozygomaticomaxillary complex in dogs. Front Vet Sci. (2023) 10:1323983. doi: 10.3389/fvets.2023.1323983, PMID: 38098991 PMC10720905

[8] Klasen JRS Thatcher GP Bleedorn JA Soukup JW. Virtual surgical planning and 3D printing: methodology and applications in veterinary oromaxillofacial surgery. Front Vet Sci. (2022) 9:971318. doi: 10.3389/fvets.2022.971318, PMID: 36337192 PMC9635215

[9] Viscasillas J Everson R Mapletoft EK Dawson C. Ultrasound-guided posterior extraconal block in the dog: anatomical study in cadavers. Vet Anaesth Analg. (2019) 46:246–50. doi: 10.1016/J.VAA.2018.09.045, PMID: 30713055

[10] Viscasillas J Ter Haar G. Ultrasound guided trigeminal nerve block as local anaesthetic technique for exenteration and excision of the zygomatic arch with partial caudal maxillectomy in a dog. Vet Anaesth Analg. (2017) 44:688–90. doi: 10.1016/J.VAA.2016.05.011 28583772

[11] Murphy CJ Samuelson DA Pollock RVH. The eye. Available at: https://scholar.google.com/scholar?hl=en&as_sdt=0%2C50&q=Murphy+CJ%2C+Samuelson+DA%2C+Pollock+RVH.+The+eye.+I&btnG (Accessed April 3, 2024).

[12] De Lott LB Kerber KA Lee PP Brown DL Burke JF. Diplopia-related ambulatory and emergency department visits in the United States, 2003-2012. JAMA Ophthalmol. (2017) 135:1339–44. doi: 10.1001/JAMAOPHTHALMOL.2017.4508, PMID: 29075739 PMC6583554

[13] Thaiwong T Sledge DG Collins-Webb A Kiupel M. Immunohistochemical characterization of canine Oral papillary squamous cell carcinoma. Vet Pathol. (2018) 55:224–32. doi: 10.1177/0300985817741732, PMID: 29262763

[14] Kuyama K Hayashi K Fufita SF Satoh I Yamamoto H. Immunohistochemical analysis of a dentigerous cyst in a dog. J Vet Dent. (2009) 26:106–9. doi: 10.1177/089875640902600205, PMID: 19718974

[15] Nemec A Murphy BG Jordan RC Kass PH Verstraete FJM. Oral papillary squamous cell carcinoma in twelve dogs. J Comp Pathol. (2014) 150:155–61. doi: 10.1016/J.JCPA.2013.07.007 24016780

[16] Tarsitano A Badiali G Pizzigallo A Marchetti C. Orbital reconstruction: patient-specific orbital floor reconstruction using a mirroring technique and a customized titanium mesh. J Craniofacial Surgery. (2016) 27:1822–5. doi: 10.1097/SCS.0000000000002907 27438454

[17] Hayes JS Richards RG. The use of titanium and stainless steel in fracture fixation. Expert Rev Med Devices. (2010) 7:843–53. doi: 10.1586/ERD.10.53 21050093

[18] Evenhuis JV Verstraete FJM Arzi B. Management of failed stainless steel implants in the oromaxillofacial region of dogs. Front Vet Sci. (2022) 9:992730. doi: 10.3389/fvets.2022.992730, PMID: 36213415 PMC9539114

[19] Vallefuoco R Bird F Gordo I Brissot H Fina C. Titanium mesh osteosynthesis for the treatment of severely comminuted maxillofacial fractures in four dogs. J Small Anim Pract. (2021) 62:903–10. doi: 10.1111/JSAP.13390 34101195

[20] Rosselli DD Platt SR Freeman C O’Neill J Kent M Holmes SP. Cranioplasty using titanium mesh after skull tumor resection in five dogs. Vet Surg. (2017) 46:67–74. doi: 10.1111/VSU.12577, PMID: 27805731

[21] Bordelon JT Rochat MC. Use of a titanium mesh for cranioplasty following radical rostrotentorial craniectomy to remove an ossifying fibroma in a dog. J Am Vet Med Assoc. (2007) 231:1692–5. doi: 10.2460/JAVMA.231.11.1692 18052805

[22] Tu T-H Thatcher GP Soukup JW. Surgical reconstruction of a composite nasomaxillary and superior labial defect in a dog with a fascia lata graft, titanium mesh implant and angularis oris axial pattern flap. Front Vet Sci. (2024) 11:1416469. doi: 10.3389/fvets.2024.1416469, PMID: 39091396 PMC11291449

[23] Gear AJL Lokeh A Aldridge JH Migliori MR Benjamin CI Schubert W. Safety of titanium mesh for orbital reconstruction. Ann Plast Surg. (2002) 48:1–9. doi: 10.1097/00000637-200201000-00001, PMID: 11773723

[24] Schubert W Gear AJL Lee C Hilger PA Haus E Migliori MR . Incorporation of titanium mesh in orbital and midface reconstruction. Plast Reconstr Surg. (2002) 110:1022–30. doi: 10.1097/01.PRS.0000021307.23118.E7 12198411

[25] Thatcher GP Soukup JW. Virtual surgical planning and 3D printing in veterinary dentistry and Oromaxillofacial surgery. Vet Clin N Am Small Anim Pract. (2022) 52:221–34. doi: 10.1016/J.CVSM.2021.09.009 34838251

[26] Winer JN Verstraete FJM Cissell DD Lucero S Athanasiou KA Arzi B. The application of 3-dimensional printing for preoperative planning in oral and maxillofacial surgery in dogs and cats. Vet Surg. (2017) 46:942–51. doi: 10.1111/VSU.12683, PMID: 28688157

